# Defining a Simplified Process in Yeast for Production of Enveloped VLP Dengue Vaccine

**DOI:** 10.3390/bioengineering12090956

**Published:** 2025-09-05

**Authors:** Salomé de Sá Magalhães, Stephen A. Morris, Shinta Kusumawardani, Acep Riza Wijayadikusumah, Neni Nurainy, Eli Keshavarz-Moore

**Affiliations:** 1 Department of Biochemical Engineering, Faculty of Engineering Sciences, University College London, London WC1E 6BT, UK; s.magalhaes@ucl.ac.uk (S.d.S.M.); stephen.morris@ucl.ac.uk (S.A.M.); 2 PT Biofarma, Bandung 40161, Indonesia; shinta.kusumawardani@biofarma.co.id (S.K.); acep.riza@biofarma.co.id (A.R.W.); nur.ainy@biofarma.co.id (N.N.); 3 Research Center for Molecular Biotechnology and Bioinformatic, University of Padjajaran, Jatinangor 45363, Indonesia

**Keywords:** dengue disease, vaccine, LMICs, *Komagataella phaffii*, VLPs, platform technology

## Abstract

Dengue is a rapidly spreading mosquito-borne viral infection, with increasing reports of outbreaks globally. According to the World Health Organization (WHO), by 30 April 2024, over 7.6 million dengue cases were reported, including 3.4 million confirmed cases, more than 16,000 severe cases, and over 3000 deaths. As dengue remains endemic in many regions, there is a critical need for the development of new vaccines and manufacturing processes that are efficient, cost-effective, and capable of meeting growing demand. In this study, we explore an alternative process development pathway for the future manufacturing of a dengue vaccine, utilizing *Komagataella phaffii* (*Pichia pastoris*) as the host organism, one of the most promising candidates for the expression of heterologous proteins in vaccine development. It combines the speed and ease of highly efficient prokaryotic platforms with some key capabilities of mammalian systems, making it ideal for scalable and cost-effective production. The key outcomes of our research include (i) demonstrating the versatility of the *Komagataella phaffii* platform in the production of dengue viral-like particles (VLPs); (ii) optimizing the culture process using Design of Experiments (DoE) approaches in small-scale bioreactors; (iii) developing a novel purification platform for enveloped VLPs (eVLPs), and (iv) establishing alternative biophysical characterization methods for the dengue vaccine prototype. These findings provide a promising foundation for efficient and scalable production of dengue vaccines, addressing both technical and operational challenges in vaccine manufacturing.

## 1. Introduction

Vaccine development remains a complex and time-consuming endeavor, constrained by lengthy clinical trial phases, stringent regulatory requirements, and challenges in large-scale manufacturing. The COVID-19 pandemic has had a profound impact on global health systems and economies, but it also catalyzed unprecedented momentum in vaccine research and development. The rapid response to SARS-CoV-2 highlighted the urgent need for adaptable, scalable vaccine platforms that can accelerate the screening and production of candidates for both emerging and endemic diseases [1,2,3].

As a result, the diversity of vaccine platforms under development has expanded considerably. Nucleic acid-based vaccines (DNA and RNA) offer advantages in speed of design and manufacture but often face limitations in immunogenicity. Subunit vaccines can be more immunologically potent but typically require complex processing and adjuvants. In contrast, virus-like particles (VLPs) have emerged as a promising alternative due to their intrinsic safety, structural mimicry of native viruses, and ability to elicit robust humoral and cellular immune responses [4,5]. These features make VLPs attractive candidates for vaccine design, particularly against infectious pathogens where immune breadth and safety are essential. While licensed virus-like particle (VLP) vaccines have not yet been approved for complex pathogens such as dengue, Ebola, and HIV, extensive preclinical and clinical research continues to demonstrate the strong immunogenic potential of VLP-based platforms. Their success in licensed vaccines against HPV and hepatitis B underscores the versatility and promise of this technology. Our study contributes to this growing body of research by exploring the feasibility of yeast-based VLP production for dengue [6,7,8].

However, further research is essential to enhance the safety profile of VLP platforms, particularly in addressing concerns related to preexisting immunity, potential genomic integration, and the emergence of virulent phenotypes. In parallel, there is an urgent need to develop effective vaccines against major human pathogens such as HIV, Epstein–Barr virus, hepatitis C virus, and tuberculosis. Strategic efforts should also focus on the development of a universal influenza vaccine and novel vectors capable of immunizing against emerging variants. Moreover, advancing multivalent vaccine formulations, expanding the use of viral vectors for passive immunization, and exploring their application in prophylactic and therapeutic cancer vaccines represent critical frontiers for future investigation.

Dengue virus is a mosquito-borne flavivirus endemic to over 100 countries, with the highest burden in Southeast Asia and the Western Pacific. The disease affects an estimated 390 million people annually, with approximately 2.5 billion currently at risk, a number projected to double by 2080 due to climate change and urbanization over [9].

The development of effective dengue vaccines is uniquely complicated by the presence of four antigenically distinct serotypes (DENV-1 to DENV-4), each capable of causing disease and eliciting serotype-specific immune responses. A major immunological challenge is the risk of antibody-dependent enhancement (ADE), wherein antibodies generated against one serotype may bind, but not neutralize, a different serotype during subsequent infection, facilitating viral entry into Fc receptor-bearing cells and exacerbating disease severity. This phenomenon has been observed both in natural infections and in clinical trials of vaccine candidates, underscoring the need for balanced and simultaneous expression of all four serotypes in vaccine prototypes [10]. Currently, two dengue vaccines: Dengvaxia^®^ (CYD-TDV) and Qdenga^®^ (TAK-003), have received regulatory approval, but both have limitations regarding age range, pre-vaccination screening requirements, and regional applicability. These limitations highlight the need for new, broadly accessible dengue vaccine platforms [11,12,13,14].

Vaccine platforms that fail to induce equivalent immunogenicity across all serotypes may inadvertently increase the risk of ADE, particularly if one or more serotypes are underrepresented or poorly immunogenic. This has been a concern in early live-attenuated tetravalent vaccines, where immunodominance of certain serotypes led to asymmetric immune responses. To address this, newer vaccine prototypes, including virus-like particle (VLP)-based and recombinant subunit approaches, are being engineered to ensure structural fidelity and antigenic balance across all four serotypes. According to literature, these platforms allow for precise control over antigen composition and presentation, reducing the likelihood of serotype bias and enhancing the potential for broad, neutralizing immunity [10,15]. However, further investigation and experimentation need to be carried out to support the findings.

Virus-like particles (VLPs) offer a compelling solution to these challenges, particularly when produced in cost-effective, scalable systems suited to Low- and Middle-Income Countries (LMICs), where dengue is most prevalent [16]. A prime example of such an approach is the use of yeast, which offers a well-established, economical, and high-yield system for producing virus-like particles (VLPs), making it particularly suitable for resource-limited settings and large-scale deployment. While it is acknowledged that yeast expression systems do not fully replicate the complex post-translational modifications (PTMs) characteristic of mammalian or insect cells, particularly in terms of glycosylation patterns that can influence protein folding and immunogenicity, yeast remains a compelling platform for vaccine development (Table 1). Its advantages include high protein yield, cost-effective cultivation, rapid growth, and ease of genetic manipulation. These features not only facilitate early-stage antigen screening and optimization but also support scalable and robust manufacturing processes [17]. Despite limitations in PTM fidelity, the proven track record of yeast in licensed vaccines (e.g., hepatitis B and HPV) underscores its value as a practical and efficient system for producing virus-like particles (VLPs) and other recombinant vaccine candidates. In our study, we explore the use of *Komagataella phaffii* (*Pichia pastoris*), a methylotrophic yeast known for its strong recombinant protein expression capacity.

*K. phaffii* combines the genetic tractability and rapid growth of prokaryotic systems with some of the post-translational modification capabilities of eukaryotic cells [22]. It has been widely used in the production of biopharmaceuticals, including monoclonal antibodies [23], enzymes, and vaccine antigens.

In recent years, *K. phaffii* has gained traction as a promising host for vaccine production in LMICs, offering a feasible route to vaccine self-sufficiency [21,24]. However, while yeast platforms are established for the production of non-enveloped VLPs, the industrial-scale production of enveloped VLPs (eVLPs) remains a technical bottleneck due to challenges in expression, purification, and product characterization [16].

In this study, we collaborated with PT Biofarma, based in Indonesia, to develop a simplified, scalable process for the intracellular expression of dengue eVLPs using *K. phaffii* as the host. Specifically, we aimed to co-express two dengue virus serotypes to evaluate the feasibility of a bivalent VLP-based vaccine prototype. Our approach focused on three key development drivers: (i) optimizing upstream fermentation through Design of Experiments (DoE) in an automated, high-throughput bioreactor system; (ii) streamlining the downstream purification process; and (iii) establishing robust biophysical characterization techniques. The overarching goal was to create a transferable VLP production platform suitable for broader use in LMICs and adaptable to other enveloped VLP-based vaccines.

## 2. Materials and Methods

Unless otherwise stated, all the chemical reagents were purchased from Sigma Aldrich (Dorset, UK)

### 2.1. Cell Line Construction and Research Cell Bank Generation

The design, selection, and transformation of the host strain were carried out by PT Biofarma (Bandung, Indonesia) using the established protocols. Briefly, codon-optimized genes encoding chimeric Dengue virus type 1 and 2 prEM/E antigens were cloned into the Pichia expression vector pAO815, with a C-terminal 6xHis tag. The recombinant plasmid was first amplified in *Escherichia coli* TOP10 (transformed by electroporation), followed by selection on Luria–Bertani (LB) agar containing ampicillin (100 μg/mL) and tetracycline (10 μg/mL). The resulting plasmid, pAO815_Den1Den2, was purified using an EndoFree Plasmid Maxi Kit (Qiagen, Germantown, MD, USA).

The pAO815_Den1Den2 plasmid was linearized with BglII (R0144S, NEB, Jakarta, Indonesia) and transformed into *Komagataella phaffii* GS115 (his4, Mut^S^) by electroporation. Selection was performed on histidine-deficient minimal dextrose (MD) plates, and methanol utilization was confirmed using minimal methanol (MM) medium. Integration of the expression cassette into the yeast genome was verified by PCR with AOX1 primers, followed by sequencing.

The selected transformants were expanded in buffered minimal glycerol (BMG) media and stored as glycerol stocks at −80 °C to establish a Research Cell Bank (RCB). Cell performance was validated through regrowth in BMG media and monitored for OD_600_ values.

### 2.2. Fermentation Process

#### Batch and Fed-Batch Cultivation

Fermentation was performed using the Ambr^®^ 250 modular microbial system (Sartorius Stedim, Stonehouse, UK) following a modified Invitrogen protocol for MutS strains [25]. Cultures were initiated in 150 mL of Basal Salt Medium (BSM) supplemented with trace salts (PTM1), at 28 °C, pH 5.0 (±0.15), and maintained at 30% dissolved oxygen (DO). The glycerol batch phase concluded upon a DO spike and a 20% decrease in the carbon evolution rate (CER).

A glycerol feed phase (18.15 mL/h/L) was followed by methanol induction using a DoE approach within the Ambr^®^250 system, modulating temperature, pH, %DO, and methanol feed rate (Table 2).

A decrease in CER during methanol adaptation indicated depletion of glycerol and was used as a signal by the system to increase methanol feed rate using an incremental step profile programmed into the Ambr^®^250 software (1 mL/h/L for 2 h and 3 mL/h/L for the remainder of the fermentation). After fermentation, the cells were harvested by centrifugation (10,000× *g*, 15 min) and stored at −20 °C.

### 2.3. Purification of Virus-like Particles (VLPs)

#### 2.3.1. Cell Lysis

Frozen cell pellets were resuspended (0.5 g/mL) in lysis buffer and disrupted by high-pressure homogenization APV Gaulin Lab40 (SPX Flow, Rochester, NY, USA) at 1200 bar for five passes (<10 °C). Lysates were clarified by centrifugation (4347× *g*, 45 min, 4 °C), and supernatants were retained.

#### 2.3.2. Sucrose Density Gradient (SDG)

Clarified lysates (2 mL) were subjected to discontinuous SDG ultracentrifugation (0–50% sucrose, 174,900 RCF, 4 h, 4 °C). Fractions were collected (1 mL), evaluated via Brix index using a MA871 refractometer (Milwaukee, Rocky Mount, NC, USA), and screened for VLP presence using dot blot (DB).

#### 2.3.3. Chromatographic Platform

The chromatography study was performed employing an AKTA Avant system (GE Healthcare Life Sciences, Cytiva, Marlborough, MA, USA), equipped with a 2 mm path length UV cell with three fixed wavelengths of 280, 260, and 320 nm. The chromatography parameters were monitored and controlled using the UNICORN 6 software (GE Healthcare Life Sciences, Cytiva, Marlborough, MA, USA). Sample homogenates were centrifuged for 30 min at 4347× *g* (max speed) and filtered with 0.45 µm polyvinylidene difluoride membrane filters (GE Healthcare Life Sciences, Buckinghamshire, UK) before loading.

##### Primary Chromatography

HisTrap™ High Performance (1 and 5 mL) from Cytiva (Marlborough, MA, USA) was performed according to the protocol provided with minor adjustments. Briefly, the column was equilibrated with 2 column volumes (CV) of 50 mM sodium phosphate, 1 mM 4-benzenesulfonyl fluoride hydrochloride (AEBSF), 5% glycerol, pH 7.4, and 20 mL of sample was injected at a flow rate of 1 mL/min. The column was washed with 4 CV of 50 mM sodium phosphate, 1 mM 4-(2-aminoethyl) benzenesulfonyl fluoride hydrochloride (AEBSF), 5% glycerol, pH 7.4, until a stable UV absorbance and conductivity were achieved. A single step elution of 5 CV of 50 mM 4-(2-Hydroxyethyl) piperazine-1-ethane-sulfonic acid (HEPES), 0.5 M NaCl, 500 mM imidazole, pH 7.4 was used for elution at 1 mL/min. Both flowthrough and elution peaks were collected in fractions of 1.5 mL. Peak fractions were pooled and subjected to a polishing step. A cleaning-in-place (CIP) procedure was performed using 0.1 M NaOH, 1 M NaCl at 1 mL/min (4 CVs).

##### Polishing Chromatography

Pooled eluted fractions from chromatography step 1 were further purified by AVIPure^TM^ Exosome (Repligen/Avitide, Waltham, MA, USA). Briefly, the column was equilibrated with 2 CV of 50 mM sodium phosphate, 1 mM 4-benzenesulfonyl fluoride hydrochloride (AEBSF), 5% glycerol, pH 7.4, and 20 mL of sample was injected at a flow rate of 1 mL/min. The column was washed with 4 CV of 50 mM sodium phosphate, 1 mM AEBSF, 5% glycerol, pH 7.4, until stable UV absorbance and conductivity were achieved. Two more washing steps were performed using 2 CV 0.5 M Tris, 50 mM HEPES, pH 7–9, and 3 CV of the buffer used for wash one. A linear gradient of 0 to 100% with 5 CV of 1 M Arginine-HCl, 50 mM HEPES, pH 9 was used for elution at 1 mL/min. Both flowthrough and elution peaks were collected in fractions of 1.5 mL and stored at 4 °C for further analysis. A cleaning-in-place (CIP) procedure was performed using 0.1 M NaOH, 1 M NaCl at 1 mL/min (4 CV).

### 2.4. Analytical Characterization

#### 2.4.1. Biochemical Assays

For SDS-PAGE assays, the samples were run on NuPAGE 4–12% Bis-Tris gels (ThermoFisher Scientific, Horsham, UK), stained with InstantBlue™, and imaged using GE Amersham™ Imager 600 (Pittsburgh, PA, USA). Following SDS-PAGE, proteins were transferred to nitrocellulose membranes and probed with mouse anti-Dengue antibody and HRP-conjugated secondary antibody, visualized using ECL substrate for Western blot (WB). For the dot blot assay, 2 μL of the sample was dotted on nitrocellulose, then blocked, incubated with primary and secondary antibodies, and imaged as per the WB protocol.

#### 2.4.2. Biophysical Assays

The samples were visualized using a MINI-TEM (Delong Instruments, Brno, Czechia), negatively stained with 2% uranyl acetate and NanoW. Grids were glow-discharged prior to sample deposition and imaging [26].

#### 2.4.3. Purity Assays

##### PicoGreen dsDNA

DNA content was quantified using the Quant-iT™ PicoGreen assay kit Life Technologies (Winsford, UK, Cat. No. P7589) with fluorescence readout at 520 nm (excitation 480 nm) using the Infinite F200 Pro plate reader (Tecan Trading AG, Männedorf, Switzerland).

##### Host Cell Protein (HCP)

Measured using the P. pastoris HCP ELISA kit (F140 Cygnus, Southport, NC, USA), absorbance at 450 nm, using the Infinite F200 Pro plate reader (Tecan Trading AG, Männedorf, Switzerland).

##### Total Protein

Protein concentration was assessed using the Pierce™ BCA Protein Assay kit (ThermoFisher Scientific, Waltham, MA, USA) with absorbance at 562 nm.

### 2.5. Statistical Analysis

Each experiment was performed at least in duplicate. The results were analyzed by one-way ANOVA and multi-comparison testing (Tukey’s HSD test), with a *p* < 0.05 considered to indicate statistical significance. All the statistical analyses were performed in Python, version 3.9.12 (Jupyter, Palm Beach County, FL, USA).

## 3. Results and Discussion

Collaborating closely with the partner company, PT Biofarma, we have pinpointed areas for development/improvement in their dengue vaccine platform. In this work, we have identified technical solutions to contribute to three critical points of vaccine manufacturing: simplicity (ease-of-doing), speed (time-efficiency), and, inherent to these, potential cost-effectiveness.

The key technical outputs achieved in this study comprise the following:

(i) Demonstration of the versatility of using the *Komagataella phaffii* platform in production of nanoparticles (dengue viral-like particles (VLPs)).
(ii) Culture optimization using DoE approaches in small-scale bioreactors.
(iii) Development of a novel VLP purification platform.
(iv) Creation of an alternative biophysical characterization of the dengue vaccine prototype.

### 3.1. Cultivation Process Improvement to Produce the Dengue VLP Vaccine Prototype (GS115/pAO815_Den1Den2)

#### 3.1.1. Host and Strain Selection

In the process, *Komagataella phaffii* (*Pichia pastoris*) was used as a host. This yeast is one of the most promising candidates for expression of heterologous proteins in vaccine development. It combines the speed and ease of processing of highly efficient prokaryotic platforms with some key capabilities of mammalian systems. Moreover, this methylotrophic yeast is an attractive option as it consolidates both high yields and protein productivity, alongside low costs, and the ability to post-translationally modify proteins [21]. Due to the way protein production is induced, i.e., the transcriptional activity of the AOX promoters in *Pichia pastoris*, the induction strategy implemented is critical. Therefore, the phenotype selection plays an important role not only in protein-to-protein interactions being expressed but also with regard to other factors, such as gene dosage [27]. In this study, we have selected the mutant Mut^S^, GS115, following current industry trends towards a reduction in methanol use, particularly at large scales. The use of this mutant allows the circumvention of issues such as the following:

(i) Safety: Methanol is highly flammable and, therefore, use at large scale is undesirable for safety reasons [28].
(ii) Oxygen demand reduction: The relationship between heat production (methanol metabolization) and oxygen consumption is linear [29]. This is important as control of reactor cooling and the transfer of oxygen are crucial to successful fermentation [30].
(iii) By-product reduction: The cytotoxicity of methanol metabolism can result in the formation of by-products such as hydrogen peroxide [31].

The clone was designed and selected by the partner company as described in the Section 2. The transformation, selection, and confirmation steps were then performed to evaluate the clones.

#### 3.1.2. Optimization of the Fermentation Process at Small Scale, Using a Design of Experiments (DoE) Approach

Investigation at the laboratory scale is a critical step for the evaluation of upstream biological processes to enable the selection of pilot-scale conditions to maximize the yield and quality of vaccine development and production [32]. With this in mind, we have combined Ambr 250^®^ experimentation with statistical Design of Experiments (DoE) as it reduces bias and the required number of experiments [33]. To improve the cultivation process, a production system where physical, chemical, or biological transformations take place can benefit from the use of mathematical tools such as DoE. In this study, the cultivation process consisted of three phases: glycerol batch, glycerol fed batch, and methanol induction phase. When producing VLPs, the induction strategy used can influence both the formation of the VLP and the product yield. Therefore, the strategy applied was to maintain the batch and fed-batch phase consistent for all runs. With that, we aimed to reduce batch variability, improve reproducibility regardless of the run week, or position of the bioreactor cell in the Ambr^®^ 250 modular. Hence, the results from the full experimental design could be attributed, with confidence, to changes in factor levels during induction (i.e., VLP production). For that, the method applied for sample randomization was a Box–Behnken design [34], where four factors (temperature, °C; pH; dissolved oxygen, %DO; and methanol feed) were considered. Factor 1 (temperature) represented 26, 28, and 30 °C; factor 2 (pH) represented 3.5, 5.0, and 6.5; factor 3 (%DO) represented 20, 30, and 40%; and factor 4 (methanol feed mL/h/L) represented 2.5, 3, and 3.5, leading to a total of 28 experiments.

**Figure 1:**
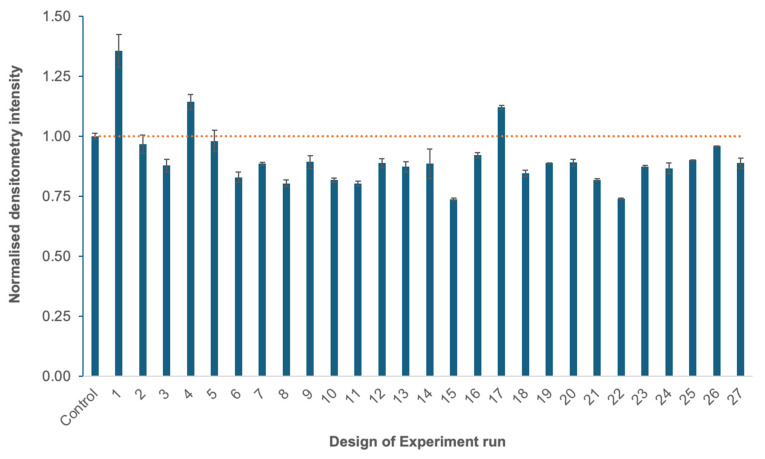
Densitometry analysis of the Box–Behnken Design of Experiment. *P. pastoris* fermentation conditions to produce vaccine candidate *GS115/pAO815_Den1Den2* were optimized by varying temperature (°C), pH, dissolved oxygen saturation (%DO), and methanol feed in Ambr^®^250 reactors. The control is the DoE central point corresponding to fermentation conditions of 28 °C, pH 5, 30 %DO, and a methanol feed of 3 mL/h/L. The densitometry results were normalized by the control (intensity 141,024 ± 1777), and experiments were performed at least in duplicates (n = 2).

At the end of the fermentation, the samples were harvested, the supernatant discharged, and the pellets subjected to HPH. Afterwards, the homogenates were subjected to SDG as a purification method. The collected fractions were then biochemically evaluated using SDS-PAGE and WB. The fractions with the given signal with anti-DENV antibody, i.e., representing the produced VLP, were then pooled and evaluated using dot blot and normalized towards the process control (central point) to facilitate the evaluation of the effect of varying factors considered during the induction phase of the fermentation process (Figure 1). The images were analyzed using the ImageQuant software (Densitometry-GE Amersham™ Imager 600 (Pittsburgh, PA, USA)).

The relative titres were then quantified and normalized towards the process control (central point), representing a set of parameters that, based on the literature [25] and previous experiments, were known to lead to the production of the target VLP. Three fermentation initial conditions produced more compared to the control, namely condition #2 (36%), #5 (14%), and #18 (12%). One-way Anova, conducted on the entire dataset, revealed a significant impact of the temperature, pH, %DO, and methanol feed on VLP production (*p* = 1.58 × 10^−13^, Table S1). Tukey’s HSD test identified that production titers at condition #2 are higher and significantly different than the control (*p* = 0.0), while conditions #5 and #18, *p* = 0.055 and *p* = 0.207, respectively, are similar (Table S2). The conditions that produce less than the control conditions are not further considered. However, these findings lay a solid foundation for future investigations that will incorporate more rigorous statistical analyses and broader experimental datasets to refine and validate the effect on different fermentation parameters on VLP production.

**Figure 2:**
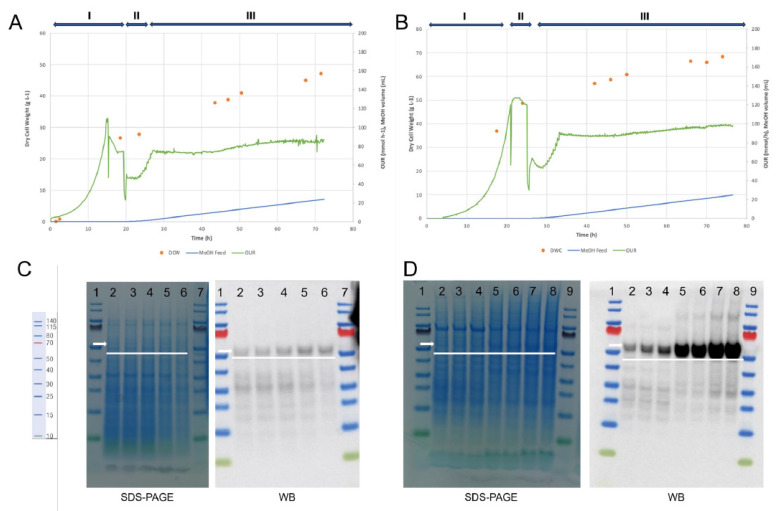
Fermentation profile and biochemical evaluation of cultivation processes: (**A**) control fermentation (benchmark) and (**B**) optimized fermentation conditions process (“DoE experiment 1”). Labels I, II, and III refer to the glycerol batch phase, glycerol fed-batch phase, and induction phase (methanol fed-batch), respectively. In orange the dry cell weight (DCM); in blue the methanol feed(MeOH Feed) and in green the oxygen uptake rate (OUR). (**C**,**D**) are the SDS-page and Western blot (WB) of the control and optimized fermentation conditions process, respectively. The first and last lane in each gel correspond to the ladder. All other lanes representing the produced VLP, purified using SDG, with a size around 55 kDa, as indicated by the white arrows.

To investigate the impact of fermentation parameters on product quality, targeted small-scale studies were performed. For clarity in interpreting Figure 2 and Figure 5, we have annotated the images with arrows indicating the expected position of the target protein bands. In the SDS-PAGE gels, the target molecule is present but appears faint, likely due to its relatively low abundance and potential co-migration with host proteins. In contrast, the Western blot clearly detects the target protein, confirming its successful expression and identity via specific antibody recognition. The discrepancy in visibility between SDS-PAGE and Western blot is consistent with the sensitivity differences between the two techniques, reflecting the higher sensitivity of Western blotting compared to Coomassie staining. These annotations and clarifications aim to improve figure readability and support the conclusions drawn from the data.

The fermentation profile of the optimized process is illustrated in Figure 2B, and its biochemical evaluation in Figure 2D using SDS-PAGE and WB, for protein profile analysis and protein specificity, respectively.

From Figure 2C,D, the product produced was present in different fractions (25–35% sucrose) with a good level of purity. Based on the intensity of the bands, improvement in the process is observable. Media, pH, and temperature had the greatest impact on the proportion of products produced. In our case, reducing pH (5 to 3.5) and temperature (28 to 26 °C) during methanol induction was observed to be a favorable strategy to increase the product titre. Reduction in pH and temperature during fermentation has been used to decrease protease activity in fermentation supernatant [35]; it was interesting to see that these parameters were the ones contributing the most to the increment in the product. However, the correlation of temperature with pH or ionic strength may be causing variable losses of structure; nonetheless, this also depends on the particle type. These results corroborate [36], where the authors described the effect of these parameters on VLP stability. Despite not being the focus of this study, an increase in induction duration (h) in comparison with the benchmark process (72 to 76 h) also seems to influence the amount of product; however, further investigation will be necessary to confirm it. This new strategy in culture growth has been technically transferred to PT Biofarma.

### 3.2. VLP Purification Process

Yeast expression systems are widely recognized for their scalability, rapid growth, and cost-effective cultivation, making them attractive platforms for vaccine production. However, the downstream purification of virus-like particles (VLPs) from yeast can present technical challenges due to the presence of host cell proteins, nucleic acids, and other process-related impurities. These contaminants often necessitate additional purification steps, such as multiple chromatographic separations and filtration, to meet regulatory standards for safety and purity [17]. Despite these complexities, advances in bioprocess engineering and purification technologies have significantly improved the efficiency and yield of VLP recovery from yeast systems [37]. Importantly, the overall cost-effectiveness of yeast-based platforms remains favorable when both upstream and downstream processes are strategically optimized. With continued innovation in purification strategies, yeast remains a highly viable and competitive alternative for the large-scale production of safe, immunogenic, and affordable VLP-based vaccines.

**Figure 3:**
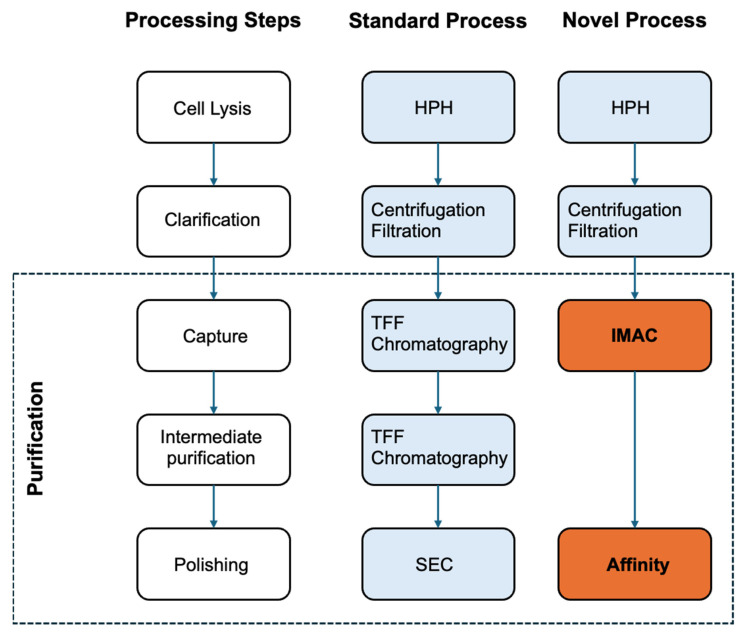
Comparison of the downstream purification process. Standard process (left-hand side) with the novel process—a simple, scalable purification procedure developed in this study (right-hand side). Purification steps were simplified by solely performing IMAC and affinity chromatography. High-pressure homogenization (HPH); tangential flow filtration (TFF); immobilized metal affinity chromatography (IMAC); and size exclusion chromatography (SEC).

To design a purification process for VLPs, aspects such as envelope presence, VLP size, propensity to aggregate, surface charge, and media composition must be accounted for [38]. Moreover, enveloped particles, as the VLPs in this study, are susceptible to shear-induced damage and vigorous clarification procedures, which may hinder intact VLP recovery [39]. Techniques, such as centrifugation, sucrose-gradient ultracentrifugation, precipitation, tangential flow filtration (TFF), and chromatography, are commonly used in downstream processing (DSP), but not all these methods are suitable for large-scale production [40]. Thus, purification is critical in VLP DSP to ensure that the specific VLPs’ physicochemical properties meet regulatory requirements. The requirements include removal of process-related impurities (e.g., host cell protein and DNA) and product-related impurities (e.g., incorrectly formed VLPs). The VLP-specific nature of these stages means that additional work can add to development times and will significantly influence ultimate large-scale manufacturing costs. Having this in mind, it was our aim to develop a VLP purification platform, which would be easy to apply and could be used for the purification of enveloped VLP in general rather than being specific to the dengue VLP used in this study. This novel purification method consists of two affinity steps: (i) based on the affinity tag incorporated in the antigenic protein, and (ii) based on the affinity of the lipid bilayer of the enveloped VLP. The first step was used as a primary purification step, where the purpose was to recover as much product of interest as possible, while the other was used as a polishing step, where only the target VLP would be recovered. Figure 3 represents an overview of the purification platform.

The clarified lysate was loaded into a HisTrap™ High Performance (5 mL), and the run was performed according to the protocol provided with minor adjustments. While His-tag affinity purification is not considered GMP-compliant due to concerns over tag-related immunogenicity, potential leaching of metal ions, and regulatory constraints on the use of genetically modified affinity tags in clinical manufacturing, it was employed in this study strictly for proof-of-concept purposes. The primary objective was to rapidly assess the feasibility and performance of the target protein under controlled experimental conditions. His-tag purification offers a well-established, high-yield, and cost-effective method for early-stage process development, enabling efficient screening of expression constructs, binding characteristics, and downstream compatibility [41]. Importantly, the insights gained from this approach inform the design of subsequent purification strategies that are fully GMP-compliant, such as tag-free affinity systems or orthogonal chromatography workflows. Thus, while not intended for clinical application, the use of His-tag purification at this stage provided critical data to support the development of a scalable and regulatory-aligned manufacturing process.

Nonetheless, this can be easily swapped by a c-tag, a viable technology for GMP-compliant manufacturing of therapeutic proteins as illustrated in [42]. Their findings support the future use of C-tag platform technologies to enable cGMP-compliant biomanufacturing of high-purity yeast-expressed VLP-based vaccines for early-phase clinical trials. That was the first use of C-tag technology to purify a VLP vaccine candidate for use in human clinical trials.

Moreover, both methods utilize affinity chromatography, meaning the core principles of the purification process remain similar, with some adjustments to the resin and elution conditions based on the specific C-tag sequence and its binding properties [43,44].

**Figure 4:**
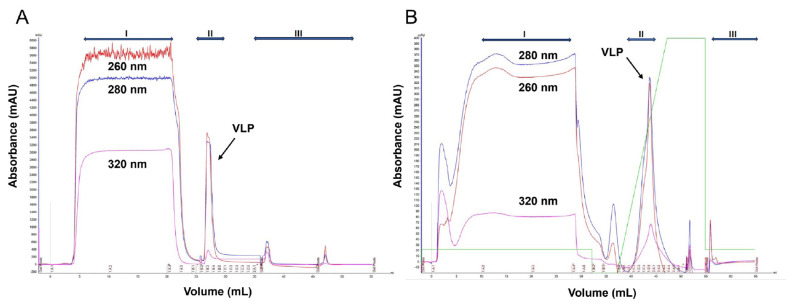
Purification of VLPs using a two-step affinity chromatography process: (**A**) primary recovery purification step using a 5 mL HisTrap™ column (IMAC) and (**B**) polishing step using a 5 mL AVIPure^TM^ column. Sections I, II, and III correspond to flowthrough, elution (in green gradient step), and clean-in-place (CIP) steps, respectively.

Afterwards, the fractions corresponding to the target VLP were pooled. This pooled sample was then diluted to minimize the interference of the imidazole on the binding capacity of the polishing step. Imidazole is known to lead to marked changes in lateral organization, curvature, and morphology of the lipid vesicles [45]. Subsequently, the diluted sample was loaded into the AVIPure^TM^ phospholipid resin and eluted using a gradient to better extract the product (Figure 4).

From the results shown in Figure 4, a two-step purification proof-of-concept was achieved, where 200 L of product can be purified using a 5 L column.

The mention of 200 L purification refers to a forward-looking projection, based on the scalability potential of the current process parameters. This projection assumes that product quality and performance can be maintained at larger volumes. The confidence in this projection is grounded in the proven robustness of the affinity chromatography workflow, originally developed for other applications but now successfully adapted for eVLP purification. The resin used in this process offers several advantages: high binding capacity, chemical stability, and compatibility with fast processing, which make it well-suited for larger-scale operations. The preliminary results from smaller-scale runs (e.g., 50 L) have shown consistent performance in terms of yield, impurity removal, and vector integrity. These findings suggest that the process can be scaled up without compromising critical quality attributes, which is especially important for clinical and commercial manufacturing where high-throughput purification is required [46]. The resin’s original use provides a strong foundation for its repurposing, offering a well-characterized performance profile and established manufacturing controls. As process optimization continues, leveraging this prior knowledge will be key to achieving reliable, GMP-compliant purification at larger scales.

Moreover, the high selectivity of the resin observed was confirmed by the specificity obtained from the biochemical evaluation of the product in the following section. Also, the A260 peak, refers to some compounds that Pichia produce that can absorb at 260 nm [47] and not to con-tamination with host cell DNA. This way, the quality of the VLP protein obtained post affinity chromatography can meet the required CQAs. This result represents the foundation for the implementation of a scalable platform for envelop VLP downstream processing. Further optimization studies are already ongoing, where for the primary purification step, we are tackling alternatives including membrane-based systems that will prove better suited for high efficiencies, while for the polishing step, a precise definition of elution and binding buffer and resins with differing ligand densities are being evaluated.

Overall, we have shown so far that our approach is effective by enhancing the upstream process with DoE and introducing a straightforward two-step purification method. As the platform evolves, further testing and refinement will be important.

### 3.3. VLP Characterization

The quality assessment of VLPs is of major importance since both physicochemical and biological properties are responsible for their clinical efficacy. The success of the VLPs as a powerful scaffold for antigen presentation and delivery strategies is dependent on the preservation of their structural integrity during all the stages of vaccine manufacturing, storage, and administration [48].

#### 3.3.1. Biochemical Characterization

**Figure 5:**
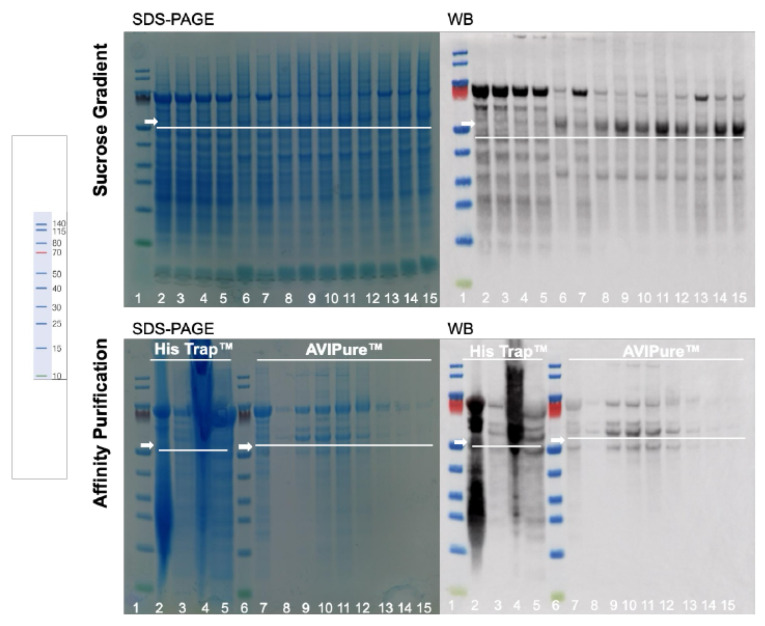
Biochemical evaluation of the produced VLP using SDS-PAGE and Western blot (WB) as characterization methods. The VLPs were purified using a sucrose gradient (benchmark), with lane 1 representing the protein ladder and all the other lanes the different fractions collected, with the target protein appearing on lines 8 to 15, with a size around 55 kDa. The VLP was also purified using affinity chromatography with different resins. Lanes 1 and 6 represent the protein ladder, lanes 2 to 5 represent purification by HisTrap™ resin, while lanes 7 to 15 are the VLP purified by the AVIPure™ resin. Lane 7 is the flowthrough, and all the other lanes are the produced VLP, with a size around 55 kDa, as indicated by the white arrows.

Following the purification strategy described in the previous section, the biochemical properties of the purified VLP were evaluated using SDS-PAGE and WB for protein profile and protein specificity, respectively (Figure 5).

The present evaluation showed that the produced protein purified by sucrose gradient, in comparison with the novel affinity chromatography method, resulted in similar protein specificity and increased purity. The two-step chromatography approach introduced here offers a notable improvement in purification efficiency, delivering protein preparations with enhanced suitability for downstream biological applications. Its streamlined design and compatibility with scale-up processes make it a practical alternative to traditional methods such as density gradient purification, with the added potential for reducing production complexity and cost.

**Figure 6:**
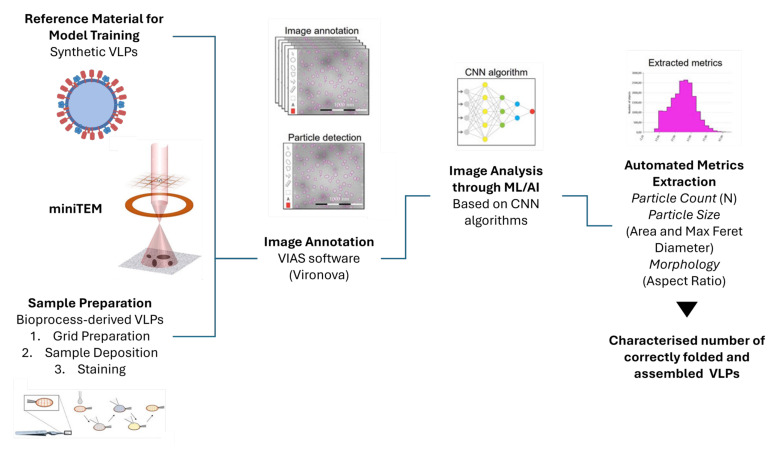
Biophysical characterization of VLP workflow using MiniTEM system. The outputs of the methodology are number of VLPs, size, and morphology. Figure adapted from [26].

As shown in Table 3, IMAC achieved over 90% removal of total protein and total DNA. However, Figure 6 reveals a persistent smear, likely due to the complexity of the sample matrix. This highlights the necessity of a polishing step to meet purity requirements. This was successfully accomplished using Avipure™, a resin specific for the selective binding of enveloped particles. The polishing step ensured high product purity, with additional impurity removal exceeding 90%.

Understanding VLP recovery yield during downstream processing is essential for assessing the feasibility of clinical or industrial applications. Low yields or complex purification processes often limit the commercial viability of otherwise valuable products. While this new methodology shows strong potential, ongoing optimization efforts aim to further enhance recovery and overall process efficiency. Past studies have demonstrated that chromatography can be applied to VLP purification, achieving higher yields than traditional ultracentrifugation methods with the advantage of being scalable [49].

Once the product purity is achieved, it is critical to evaluate its specificity. Even though the vaccine prototype being analyzed in this study only targets DENV1 and DENV2, as mentioned previously, the ultimate goal will be the creation of a multivalent vaccine against the four dengue serotypes. Therefore, it is critical to understand the expression ratio of the proteins. Based on this, we have analyzed it for this specific prototype (Table 4).

The proportion of the serotypes is maintained independently of the type of affinity chromatography. Our current results indicate that the proportion of DENV1 is higher than DENV2 across both affinity chromatography strategies, and this trend appears to be independent of the purification method used. While this could be attributed to the expression system inherently favoring DENV1 expression, it is also plausible that both serotypes are expressed at similar levels, but DENV1 is more efficiently incorporated into the VLPs. Further analysis will have to be performed to understand in depth the effect observed.

Nevertheless, this result gives valuable insights regarding the product quality throughout the process. Furthermore, it can be crucial information to be used in formulation concerning serotype balancing to avoid ADE effect, and that way achieve a multivalent vaccine.

The final samples were evaluated according to purity and quality (Table 5). We achieved a final concentration of 118 ng/mL for DENV 1 and 70 ng/mL for DENV2, with 2.7 μg/mL HCP and 81 ng/mL of total DNA as impurities.

At this early stage of development, we have not yet fully elucidated the mechanisms underlying this imbalance. Further in-depth analyses, such as quantitative assessments of intracellular protein expression levels and VLP assembly efficiency, will be necessary to determine whether the observed serotype distribution stems from differential expression or preferential packaging.

This study establishes *K. phaffii* as a promising host for the production of enveloped virus-like particles (VLPs), with initial characterization performed using biophysical and biochemical methods. These early insights contribute to our understanding of product quality across the production process and highlight the importance of achieving a balanced serotype formulation, particularly to mitigate the risk of antibody-dependent enhancement (ADE), a known challenge in dengue vaccine development. Although functional validation, such as measurements of neutralizing antibody titers or in vivo protection, was beyond the scope of this initial investigation, these data are essential for evaluating vaccine efficacy and safety. As the platform advances, transitioning to a tetravalent formulation and conducting preclinical studies in relevant animal models will be the critical next steps. Such efforts will build upon the foundation laid here, supporting the continued optimization and development of a safe, effective, and scalable multivalent VLP-based vaccine candidate.

Nonetheless, this biochemical evaluation cannot give us information regarding structures assembled from non-assembled structures [50]. Ensuring the structural fidelity of virus-like particles (VLPs) is essential for their ability to elicit a protective immune response. Accurate mimicry of the native virus morphology is critical for preserving conformational epitopes that are necessary for effective immune recognition and neutralizing antibody production [7]. To confirm this structural integrity, it is recommended to employ high-resolution characterization techniques such as transmission electron microscopy (TEM), cryo-electron microscopy (cryo-EM), and dynamic light scattering (DLS), which allow for detailed assessment of particle size, shape, and uniformity [7,51]. In parallel, immunogenicity studies, both in vitro and in vivo, are necessary to validate the functional relevance of the VLPs and to ensure that structural features translate into robust immune activation [52]. These combined approaches provide a comprehensive framework for evaluating the quality and efficacy of VLP-based vaccine candidates.

#### 3.3.2. Biophysical Characterization

Transmission electron microscopy (TEM) plays a key role in defining quality control criteria during process development and manufacturing. This methodology remains a gold standard for the structural characterization of virus-like particles (VLPs), offering high-resolution visualization of particle morphology, size, and integrity; however, the use of TEM as a routine analytical tool in manufacturing settings is hampered by several factors, such as complex sample preparation, specific facilities, and the need for highly trained staff. We developed a methodology using a new system known as MiniTEM [26], establishing a novel way to characterize the physical traits of the VLPs.

The MiniTEM system, a compact, automated version of conventional TEM, has emerged as a powerful tool for routine, high-throughput analysis of VLPs and other nanoparticle-based biologics. Its integration of automated image acquisition and machine learning-based particle classification enables rapid, objective, and reproducible assessments of critical quality attributes such as particle size distribution, shape uniformity, and aggregation state. This is particularly valuable in vaccine development, where maintaining structural fidelity is essential for preserving immunogenic epitopes. The user-friendly interface and minimal infrastructure requirements of MiniTEM make it a practical alternative to traditional TEM, especially in environments where speed, consistency, and scalability are critical [26,53]. To support the reliable and efficient application of transmission electron microscopy (TEM) for analyzing nano-scale biological materials in Biosafety Level 2 (BSL2) environments, a structured and robust workflow was established. This framework addresses the specific operational demands of BSL2 laboratories, such as adherence to strict safety measures, thorough equipment decontamination, and the involvement of appropriately trained staff. A compact, low-voltage TEM system (MiniTEM™) was integrated to enable high-throughput, automated imaging in these settings, aligning with regulatory expectations for detailed morphological characterization in vaccine and gene therapy research [54].

Another example of the utility of MiniTEM for VLP characterization was demonstrated by Ryner et al. [53], who used the system to monitor influenza VLP integrity and purity during downstream processing. Through automated image analysis, MiniTEM distinguished VLPs from impurities and quantified hemagglutinin (HA) spikes on their surface. This approach confirmed that HA detected by conventional assays was associated with intact VLPs, not free protein or debris, and showed that spike density was maintained throughout processing. These findings highlight MiniTEM as a valuable orthogonal tool for structural assessment and process monitoring in VLP-based vaccine development [53].

Moreover, the integration of artificial intelligence (AI) into VLP research has catalyzed significant advancements across the development pipeline. In the development stage, AI-driven models are increasingly used to optimize upstream parameters such as culture conditions, nutrient concentrations, and growth kinetics, enhancing yield and consistency. In structural analysis, AI accelerates image processing and particle recognition in electron microscopy, enabling faster and more accurate quantification of VLP populations. Additionally, predictive modeling using machine learning algorithms supports the forecasting of VLP stability and behavior under various conditions, guiding formulation and storage strategies. Together, these innovations position AI and MiniTEM as complementary technologies that may significantly enhance the precision, efficiency, and scalability of VLP-based vaccine development [55].

Our study was the result of the greater demand for high-throughput, rapid analytics to determine in real time the impact of in-process changes on final product quality. The study is summarized in Figure 6 and comprises two main phases: firstly, sample/grid prep optimization through the development of a protocol establishing clear guidelines/methods for preparing VLPs using non-radioactive stains for TEM analysis, and secondly, and building on the former, the generation of a methodology with quality acceptance criteria for the analysis of VLPs and viral vectors in manufacturing settings using a MiniTEM system as an analytical tool.

We then applied the developed method [26] to determine the biophysical characteristics of the produced VLP (Figure 6).

**Figure 7:**
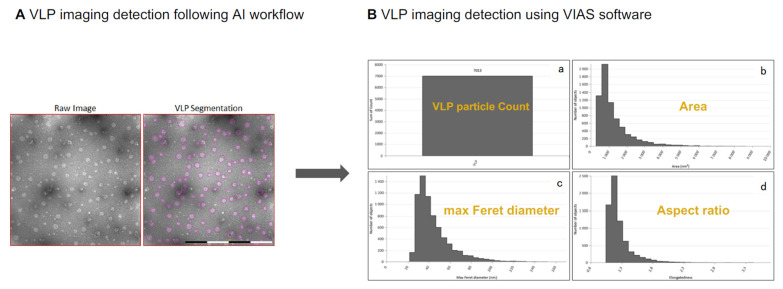
Biophysical evaluation of the produced VLP using the established workflow. Images taken by the MiniTEM system are analyzed using proprietary machine learning approaches, i.e., Vironova Imaging and Analysis Software (VIAS), a TEM-based image analysis tool for nanoparticles, compliant with FDA 21 CFR Part 11. Figure adapted from [26].

Figure 7A shows the application of the created method to the images and the automated particle detection performance evaluation. The pink circles show that the method was able to successfully differentiate between VLP, background, and debris, providing a flexible and tunable model for future studies and analyses. On the other hand and taking advantage of the capability of the AI models used, the best fit between input data and predicted image outcome was measured (Figure 7B). The statistical analysis showed that the model detected around seven thousand particles of interest (VLPs). Those particles presented an average area equivalent to a 32 nm circle, matching a max ferret diameter ranging from 30 to 40 nm, which is in accordance with the VLP size as reported in the literature [41], using orthogonal measurements, and an aspect ratio around 1, in accordance with the common circular shape of the VLP.

This methodology can therefore provide a user-friendly and fast way to characterize VLPs without the need for the currently available TEM [26].

With its small footprint and semi-automated workflows, MiniTEM is well-suited for use in manufacturing environments where space, consistency, and speed are critical. Its built-in deep learning algorithms (convolutional neural networks) enable automated image analysis, reducing reliance on expert operators and significantly increasing the throughput and consistency of data interpretation. This automation facilitates the processing of larger datasets, aligning with the needs of industrial-scale production. Further validation is needed for full GMP integration. This work broadens the utility of MiniTEM beyond its established role in orthogonal particle characterization, positioning it as a valuable tool for biologics development and quality control in resource-limited settings [56,57,58].

Its adaptability, ease of use, and data-driven capabilities position it well for routine monitoring of product quality and support decision-making throughout the vaccine development and production lifecycle.

## 4. Conclusions

This study highlights the feasibility and promises of a yeast-based platform for the production of enveloped virus-like particles (eVLPs) as dengue vaccine candidates. Using *Komagataella phaffii*, we successfully co-expressed DENV1 and DENV2 as a bivalent prototype and established a flexible, scalable upstream process. Through a design of experiments (DoE) approach, we identified optimal fermentation conditions, while a streamlined two-step purification strategy featuring a repurposed phospholipid-based resin enabled high purity and yield with reduced complexity [46]. These innovations offer a cost-effective and potentially GMP-compatible alternative to traditional ultracentrifugation-based methods.

Our findings are well aligned with recent advancements in recombinant technology that position Pichia pastoris as a powerful alternative to mammalian and insect cell systems. Enhanced scalability, cost-efficiency, and post-translational capabilities make yeast-based platforms particularly attractive for vaccine development in resource-limited settings [59]. Optimized fermentation and purification strategies have already demonstrated consistent VLP yields exceeding 1 μg/mL without compromising antigenic integrity [60], and recent preclinical and clinical data further validate the translational potential of yeast-derived dengue VLPs [61]. Notably, a tetravalent candidate has shown robust immunogenicity across all four serotypes with no evidence of antibody-dependent enhancement (ADE), a key safety milestone [62].

Together, these developments underscore the transformative potential of yeast-based platforms in overcoming key bottlenecks in dengue vaccine manufacturing. While this study focused on a bivalent prototype, it lays a strong foundation for the development of a tetravalent formulation and broader application to other VLP-based vaccines. This work contributes to a growing body of evidence supporting scalable, affordable, and globally accessible immunization strategies, particularly critical for low- and middle-income countries facing the dual challenges of endemic disease and limited manufacturing infrastructure.

## Figures and Tables

**Table 1:** Comparison table summarizing the key differences between yeast and insect cell expression systems for VLP vaccine production, with a focus on post-translational modifications (PTMs) and other relevant factors [18,19,20,21].

Feature	Yeast (*Komagataella phaffii*)	Insect Cells (e.g., Sf9, High Five)
**Growth rate**	Fast	Moderate
**Cost of cultivation**	Low	Higher
**Scalability**	High	Moderate
**PTM fidelity**	Limited (e.g., hypermannosylation)	Better than yeast, but not fully human-like
**Glycosylation**	High mannose-type, lacks sialylation	Complex-type, lacks terminal sialic acids
**Protein folding**	May be suboptimal for complex proteins	Better suited for complex protein folding
**Immunogenicity of VLPs**	Often strong, but PTMs may affect epitopes	Strong, with a more native-like antigen structure
**Regulatory acceptance**	Established (e.g., HBV, HPV vaccines)	Increasing, used in licensed vaccines (e.g., FluBlok)
**Production yield**	High	Moderate
**Use in licensed vaccines**	Yes (e.g., HBV, HPV)	Yes (e.g., FluBlok for influenza)

**Table 2:** Levels of variables tested in the Box–Behnken design for the eVLP production optimization.

Variables	Levels		
	−1 (min.)	0	1 (max.)
Temperature (°C)	26	28	30
pH (-)	3.5	5.0	6.5
Dissolved oxygen (%)	20	30	40
Methanol feed (mL/h/L)	2.5	3	3.5

**Table 3:** Impurity removal from VLP samples using different chromatography methods.

Chromatography Method	Removal Yield (%)	
	Total Protein	Total DNA
Histrap™	96 ± 0.1	97 ± 3.0
Avipure™	94 ± 0.03	93 ± 1.0

**Table 4:** Individual serotype proportion in comparison with total serotype expression using different chromatography methods.

Chromatography Method	Proportion %	
	Ratio DENV1	Ratio DENV2
Histrap™	57 ± 0.02	43 ± 0.02
Avipure™	62 ± 0.04	38 ± 0.04

**Table 5:** Concentration values of the different evaluated parameters, namely serotypes DENV1 and DENV2, host cell protein (HCP), and total DNA.

DENV1 (ng/mL)	DENV2 (ng/mL)	HCP (μg/mL)	Total DNA (ng/mL)
118 ± 3.0	70 ± 2.0	2.7 ± 0.4	81 ± 3.0

## Data Availability

The original contributions presented in this study are included in the article/Supplementary Material. Further inquiries can be directed to the corresponding author.

## Supplementary Materials

The following supporting information can be downloaded at: https://www.mdpi.com/article/10.3390/bioengineering12090956/s1, Table S1: One-way ANOVA results of densitometry analysis. Comparison of fermentation conditions and impact on vaccine candidate *GS115/pAO815_Den1Den2* production. Table S2: Tukey’s HSD test results for pairwise comparison of fermentation conditions and impact on vaccine candidate *GS115/pAO815_Den1Den2* production.
