# Supplementary material for: Defining a Simplified Process in Yeast for Production of Enveloped VLP Dengue Vaccine
Source: Bioengineering (Basel). 2025 Sep 5;12(9):956. doi: 10.3390/bioengineering12090956 (PMC12467798; doi:10.3390/bioengineering12090956)
Supplement: Supplementary file 1 [file bioengineering-12-00956-s001.zip › bioengineering-3658033-supplementary.pdf]

Supplementary Material

Defining a simplified process in yeast for production of enveloped VLP Dengue vaccine

Salome de Sa Magalhaes[1], Stephen A. Morris[1], Shinta Kusumawardani[2,3], Acep Riza Wijayadikusumah[2], Neni Nurainy[2] and Eli Keshavarz-Moore [1,*]

[1] Dept of Biochemical Engineering, Faculty of Engineering Sciences, UCL, UK;
[2] PT Biofarma, Bandung, Indonesia;
[3] Research Center for Molecular Biotechnology and Bioinformatic, University of Padjajaran

Table S1: One-way ANOVA results of densitometry analysis. Comparison of fermentation conditions and impact on vaccine candidate *GS115/pAO815_Den1Den2* production.

	Sum_sq	df	F	PR (>F)	Comment
**Experiment**	1.73×10^{10}	27	25.65	1.58×10^{-13}	Significant
**Residual**	6.99×10^{8}	28	NaN	NaN	

Table S2: Tukey's HSD test results for pairwise comparison of fermentation conditions and impact on vaccine candidate *GS115/pAO815_Den1Den2* production.

Group 1	Group 2	Mean diff	p-adj	Lower	Upper	Reject
1	2	50330.5	0	29804.818	70856.182	TRUE
1	3	-4461	1	-24986.682	16064.682	FALSE
1	4	-17032.5	0.2103	-37558.182	3493.1819	FALSE
1	5	20310.5	0.0551	-215.1819	40836.182	FALSE
1	6	-2568	1	-23093.682	17957.682	FALSE
1	7	-24011.5	0.0096	-44537.182	-3485.8181	TRUE
1	8	-15942.5	0.3065	-36468.182	4583.1819	FALSE
1	9	-27497.5	0.0017	-48023.182	-6971.8181	TRUE
1	10	-14958.5	0.4143	-35484.182	5567.1819	FALSE
1	11	-25674.5	0.0042	-46200.182	-5148.8181	TRUE
1	12	-27771.5	0.0014	-48297.182	-7245.8181	TRUE
1	13	-15654.5	0.3361	-36180.182	4871.1819	FALSE
1	14	-17728.5	0.162	-38254.182	2797.1819	FALSE
1	15	-16080	0.293	-36605.682	4445.6819	FALSE
1	16	-36943.5	0	-57469.182	-16417.818	TRUE
1	17	-10983	0.883	-31508.682	9542.6819	FALSE
1	18	17067	0.2077	-3458.6819	37592.682	FALSE

1	19	-21646.5	0.0299	-42172.182	-1120.8181	TRUE
1	20	-15423.5	0.3611	-35949.182	5102.1819	FALSE
1	21	-15337	0.3707	-35862.682	5188.6819	FALSE
1	22	-25460.5	0.0047	-45986.182	-4934.8181	TRUE
1	23	-36610	0	-57135.682	-16084.318	TRUE
1	24	-17566	0.1724	-38091.682	2959.6819	FALSE
1	25	-18676.5	0.111	-39202.182	1849.1819	FALSE
1	26	-13894	0.5475	-34419.682	6631.6819	FALSE
1	27	-5681.5	1	-26207.182	14844.182	FALSE
1	28	-15651.5	0.3364	-36177.182	4874.1819	FALSE
2	3	-54791.5	0	-75317.182	-34265.818	TRUE
2	4	-67363	0	-87888.682	-46837.318	TRUE
2	5	-30020	0.0005	-50545.682	-9494.3181	TRUE
2	6	-52898.5	0	-73424.182	-32372.818	TRUE
2	7	-74342	0	-94867.682	-53816.318	TRUE
2	8	-66273	0	-86798.682	-45747.318	TRUE
2	9	-77828	0	-98353.682	-57302.318	TRUE
2	10	-65289	0	-85814.682	-44763.318	TRUE
2	11	-76005	0	-96530.682	-55479.318	TRUE
2	12	-78102	0	-98627.682	-57576.318	TRUE
2	13	-65985	0	-86510.682	-45459.318	TRUE
2	14	-68059	0	-88584.682	-47533.318	TRUE
2	15	-66410.5	0	-86936.182	-45884.818	TRUE
2	16	-87274	0	-107799.68	-66748.318	TRUE
2	17	-61313.5	0	-81839.182	-40787.818	TRUE
2	18	-33263.5	0.0001	-53789.182	-12737.818	TRUE
2	19	-71977	0	-92502.682	-51451.318	TRUE
2	20	-65754	0	-86279.682	-45228.318	TRUE
2	21	-65667.5	0	-86193.182	-45141.818	TRUE
2	22	-75791	0	-96316.682	-55265.318	TRUE
2	23	-86940.5	0	-107466.18	-66414.818	TRUE
2	24	-67896.5	0	-88422.182	-47370.818	TRUE
2	25	-69007	0	-89532.682	-48481.318	TRUE
2	26	-64224.5	0	-84750.182	-43698.818	TRUE
2	27	-56012	0	-76537.682	-35486.318	TRUE
2	28	-65982	0	-86507.682	-45456.318	TRUE
3	4	-12571.5	0.7173	-33097.182	7954.1819	FALSE
3	5	24771.5	0.0066	4245.8181	45297.182	TRUE
3	6	1893	1	-18632.682	22418.682	FALSE
3	7	-19550.5	0.0768	-40076.182	975.1819	FALSE
3	8	-11481.5	0.8384	-32007.182	9044.1819	FALSE
3	9	-23036.5	0.0154	-43562.182	-2510.8181	TRUE

3	10	-10497.5	0.9185	-31023.182	10028.182	FALSE
3	11	-21213.5	0.0366	-41739.182	-687.8181	TRUE
3	12	-23310.5	0.0135	-43836.182	-2784.8181	TRUE
3	13	-11193.5	0.8651	-31719.182	9332.1819	FALSE
3	14	-13267.5	0.6291	-33793.182	7258.1819	FALSE
3	15	-11619	0.8248	-32144.682	8906.6819	FALSE
3	16	-32482.5	0.0001	-53008.182	-11956.818	TRUE
3	17	-6522	0.9998	-27047.682	14003.682	FALSE
3	18	21528	0.0316	1002.3181	42053.682	TRUE
3	19	-17185.5	0.1988	-37711.182	3340.1819	FALSE
3	20	-10962.5	0.8846	-31488.182	9563.1819	FALSE
3	21	-10876	0.8915	-31401.682	9649.6819	FALSE
3	22	-20999.5	0.0403	-41525.182	-473.8181	TRUE
3	23	-32149	0.0002	-52674.682	-11623.318	TRUE
3	24	-13105	0.6501	-33630.682	7420.6819	FALSE
3	25	-14215.5	0.5061	-34741.182	6310.1819	FALSE
3	26	-9433	0.9699	-29958.682	11092.682	FALSE
3	27	-1220.5	1	-21746.182	19305.182	FALSE
3	28	-11190.5	0.8654	-31716.182	9335.1819	FALSE
4	5	37343	0	16817.318	57868.682	TRUE
4	6	14464.5	0.4746	-6061.1819	34990.182	FALSE
4	7	-6979	0.9994	-27504.682	13546.682	FALSE
4	8	1090	1	-19435.682	21615.682	FALSE
4	9	-10465	0.9206	-30990.682	10060.682	FALSE
4	10	2074	1	-18451.682	22599.682	FALSE
4	11	-8642	0.9885	-29167.682	11883.682	FALSE
4	12	-10739	0.9018	-31264.682	9786.6819	FALSE
4	13	1378	1	-19147.682	21903.682	FALSE
4	14	-696	1	-21221.682	19829.682	FALSE
4	15	952.5	1	-19573.182	21478.182	FALSE
4	16	-19911	0.0657	-40436.682	614.6819	FALSE
4	17	6049.5	0.9999	-14476.182	26575.182	FALSE
4	18	34099.5	0.0001	13573.818	54625.182	TRUE
4	19	-4614	1	-25139.682	15911.682	FALSE
4	20	1609	1	-18916.682	22134.682	FALSE
4	21	1695.5	1	-18830.182	22221.182	FALSE
4	22	-8428	0.9914	-28953.682	12097.682	FALSE
4	23	-19577.5	0.0759	-40103.182	948.1819	FALSE
4	24	-533.5	1	-21059.182	19992.182	FALSE
4	25	-1644	1	-22169.682	18881.682	FALSE
4	26	3138.5	1	-17387.182	23664.182	FALSE
4	27	11351	0.8508	-9174.6819	31876.682	FALSE

4	28	1381	1	-19144.682	21906.682	FALSE
5	6	-22878.5	0.0167	-43404.182	-2352.8181	TRUE
5	7	-44322	0	-64847.682	-23796.318	TRUE
5	8	-36253	0	-56778.682	-15727.318	TRUE
5	9	-47808	0	-68333.682	-27282.318	TRUE
5	10	-35269	0	-55794.682	-14743.318	TRUE
5	11	-45985	0	-66510.682	-25459.318	TRUE
5	12	-48082	0	-68607.682	-27556.318	TRUE
5	13	-35965	0	-56490.682	-15439.318	TRUE
5	14	-38039	0	-58564.682	-17513.318	TRUE
5	15	-36390.5	0	-56916.182	-15864.818	TRUE
5	16	-57254	0	-77779.682	-36728.318	TRUE
5	17	-31293.5	0.0002	-51819.182	-10767.818	TRUE
5	18	-3243.5	1	-23769.182	17282.182	FALSE
5	19	-41957	0	-62482.682	-21431.318	TRUE
5	20	-35734	0	-56259.682	-15208.318	TRUE
5	21	-35647.5	0	-56173.182	-15121.818	TRUE
5	22	-45771	0	-66296.682	-25245.318	TRUE
5	23	-56920.5	0	-77446.182	-36394.818	TRUE
5	24	-37876.5	0	-58402.182	-17350.818	TRUE
5	25	-38987	0	-59512.682	-18461.318	TRUE
5	26	-34204.5	0.0001	-54730.182	-13678.818	TRUE
5	27	-25992	0.0036	-46517.682	-5466.3181	TRUE
5	28	-35962	0	-56487.682	-15436.318	TRUE
6	7	-21443.5	0.0329	-41969.182	-917.8181	TRUE
6	8	-13374.5	0.6152	-33900.182	7151.1819	FALSE
6	9	-24929.5	0.0061	-45455.182	-4403.8181	TRUE
6	10	-12390.5	0.7393	-32916.182	8135.1819	FALSE
6	11	-23106.5	0.0149	-43632.182	-2580.8181	TRUE
6	12	-25203.5	0.0053	-45729.182	-4677.8181	TRUE
6	13	-13086.5	0.6525	-33612.182	7439.1819	FALSE
6	14	-15160.5	0.3907	-35686.182	5365.1819	FALSE
6	15	-13512	0.5973	-34037.682	7013.6819	FALSE
6	16	-34375.5	0	-54901.182	-13849.818	TRUE
6	17	-8415	0.9916	-28940.682	12110.682	FALSE
6	18	19635	0.0741	-890.6819	40160.682	FALSE
6	19	-19078.5	0.0939	-39604.182	1447.1819	FALSE
6	20	-12855.5	0.682	-33381.182	7670.1819	FALSE
6	21	-12769	0.6929	-33294.682	7756.6819	FALSE
6	22	-22892.5	0.0165	-43418.182	-2366.8181	TRUE
6	23	-34042	0.0001	-54567.682	-13516.318	TRUE
6	24	-14998	0.4096	-35523.682	5527.6819	FALSE

6	25	-16108.5	0.2902	-36634.182	4417.1819	FALSE
6	26	-11326	0.8532	-31851.682	9199.6819	FALSE
6	27	-3113.5	1	-23639.182	17412.182	FALSE
6	28	-13083.5	0.6529	-33609.182	7442.1819	FALSE
7	8	8069	0.995	-12456.682	28594.682	FALSE
7	9	-3486	1	-24011.682	17039.682	FALSE
7	10	9053	0.9805	-11472.682	29578.682	FALSE
7	11	-1663	1	-22188.682	18862.682	FALSE
7	12	-3760	1	-24285.682	16765.682	FALSE
7	13	8357	0.9923	-12168.682	28882.682	FALSE
7	14	6283	0.9999	-14242.682	26808.682	FALSE
7	15	7931.5	0.996	-12594.182	28457.182	FALSE
7	16	-12932	0.6723	-33457.682	7593.6819	FALSE
7	17	13028.5	0.6599	-7497.1819	33554.182	FALSE
7	18	41078.5	0	20552.818	61604.182	TRUE
7	19	2365	1	-18160.682	22890.682	FALSE
7	20	8588	0.9893	-11937.682	29113.682	FALSE
7	21	8674.5	0.988	-11851.182	29200.182	FALSE
7	22	-1449	1	-21974.682	19076.682	FALSE
7	23	-12598.5	0.714	-33124.182	7927.1819	FALSE
7	24	6445.5	0.9998	-14080.182	26971.182	FALSE
7	25	5335	1	-15190.682	25860.682	FALSE
7	26	10117.5	0.9409	-10408.182	30643.182	FALSE
7	27	18330	0.1278	-2195.6819	38855.682	FALSE
7	28	8360	0.9923	-12165.682	28885.682	FALSE
8	9	-11555	0.8312	-32080.682	8970.6819	FALSE
8	10	984	1	-19541.682	21509.682	FALSE
8	11	-9732	0.9589	-30257.682	10793.682	FALSE
8	12	-11829	0.803	-32354.682	8696.6819	FALSE
8	13	288	1	-20237.682	20813.682	FALSE
8	14	-1786	1	-22311.682	18739.682	FALSE
8	15	-137.5	1	-20663.182	20388.182	FALSE
8	16	-21001	0.0403	-41526.682	-475.3181	TRUE
8	17	4959.5	1	-15566.182	25485.182	FALSE
8	18	33009.5	0.0001	12483.818	53535.182	TRUE
8	19	-5704	1	-26229.682	14821.682	FALSE
8	20	519	1	-20006.682	21044.682	FALSE
8	21	605.5	1	-19920.182	21131.182	FALSE
8	22	-9518	0.967	-30043.682	11007.682	FALSE
8	23	-20667.5	0.0469	-41193.182	-141.8181	TRUE
8	24	-1623.5	1	-22149.182	18902.182	FALSE
8	25	-2734	1	-23259.682	17791.682	FALSE

8	26	2048.5	1	-18477.182	22574.182	FALSE
8	27	10261	0.933	-10264.682	30786.682	FALSE
8	28	291	1	-20234.682	20816.682	FALSE
9	10	12539	0.7213	-7986.6819	33064.682	FALSE
9	11	1823	1	-18702.682	22348.682	FALSE
9	12	-274	1	-20799.682	20251.682	FALSE
9	13	11843	0.8015	-8682.6819	32368.682	FALSE
9	14	9769	0.9574	-10756.682	30294.682	FALSE
9	15	11417.5	0.8446	-9108.1819	31943.182	FALSE
9	16	-9446	0.9694	-29971.682	11079.682	FALSE
9	17	16514.5	0.2528	-4011.1819	37040.182	FALSE
9	18	44564.5	0	24038.818	65090.182	TRUE
9	19	5851	1	-14674.682	26376.682	FALSE
9	20	12074	0.776	-8451.6819	32599.682	FALSE
9	21	12160.5	0.7662	-8365.1819	32686.182	FALSE
9	22	2037	1	-18488.682	22562.682	FALSE
9	23	-9112.5	0.9791	-29638.182	11413.182	FALSE
9	24	9931.5	0.9502	-10594.182	30457.182	FALSE
9	25	8821	0.9854	-11704.682	29346.682	FALSE
9	26	13603.5	0.5853	-6922.1819	34129.182	FALSE
9	27	21816	0.0276	1290.3181	42341.682	TRUE
9	28	11846	0.8011	-8679.6819	32371.682	FALSE
10	11	-10716	0.9035	-31241.682	9809.6819	FALSE
10	12	-12813	0.6873	-33338.682	7712.6819	FALSE
10	13	-696	1	-21221.682	19829.682	FALSE
10	14	-2770	1	-23295.682	17755.682	FALSE
10	15	-1121.5	1	-21647.182	19404.182	FALSE
10	16	-21985	0.0255	-42510.682	-1459.3181	TRUE
10	17	3975.5	1	-16550.182	24501.182	FALSE
10	18	32025.5	0.0002	11499.818	52551.182	TRUE
10	19	-6688	0.9997	-27213.682	13837.682	FALSE
10	20	-465	1	-20990.682	20060.682	FALSE
10	21	-378.5	1	-20904.182	20147.182	FALSE
10	22	-10502	0.9182	-31027.682	10023.682	FALSE
10	23	-21651.5	0.0298	-42177.182	-1125.8181	TRUE
10	24	-2607.5	1	-23133.182	17918.182	FALSE
10	25	-3718	1	-24243.682	16807.682	FALSE
10	26	1064.5	1	-19461.182	21590.182	FALSE
10	27	9277	0.9747	-11248.682	29802.682	FALSE
10	28	-693	1	-21218.682	19832.682	FALSE
11	12	-2097	1	-22622.682	18428.682	FALSE
11	13	10020	0.9459	-10505.682	30545.682	FALSE

11	14	7946	0.9959	-12579.682	28471.682	FALSE
11	15	9594.5	0.9643	-10931.182	30120.182	FALSE
11	16	-11269	0.8584	-31794.682	9256.6819	FALSE
11	17	14691.5	0.4464	-5834.1819	35217.182	FALSE
11	18	42741.5	0	22215.818	63267.182	TRUE
11	19	4028	1	-16497.682	24553.682	FALSE
11	20	10251	0.9336	-10274.682	30776.682	FALSE
11	21	10337.5	0.9285	-10188.182	30863.182	FALSE
11	22	214	1	-20311.682	20739.682	FALSE
11	23	-10935.5	0.8868	-31461.182	9590.1819	FALSE
11	24	8108.5	0.9947	-12417.182	28634.182	FALSE
11	25	6998	0.9993	-13527.682	27523.682	FALSE
11	26	11780.5	0.8081	-8745.1819	32306.182	FALSE
11	27	19993	0.0634	-532.6819	40518.682	FALSE
11	28	10023	0.9457	-10502.682	30548.682	FALSE
12	13	12117	0.7712	-8408.6819	32642.682	FALSE
12	14	10043	0.9447	-10482.682	30568.682	FALSE
12	15	11691.5	0.8174	-8834.1819	32217.182	FALSE
12	16	-9172	0.9775	-29697.682	11353.682	FALSE
12	17	16788.5	0.2296	-3737.1819	37314.182	FALSE
12	18	44838.5	0	24312.818	65364.182	TRUE
12	19	6125	0.9999	-14400.682	26650.682	FALSE
12	20	12348	0.7443	-8177.6819	32873.682	FALSE
12	21	12434.5	0.734	-8091.1819	32960.182	FALSE
12	22	2311	1	-18214.682	22836.682	FALSE
12	23	-8838.5	0.9851	-29364.182	11687.182	FALSE
12	24	10205.5	0.9361	-10320.182	30731.182	FALSE
12	25	9095	0.9795	-11430.682	29620.682	FALSE
12	26	13877.5	0.5496	-6648.1819	34403.182	FALSE
12	27	22090	0.0243	1564.3181	42615.682	TRUE
12	28	12120	0.7708	-8405.6819	32645.682	FALSE
13	14	-2074	1	-22599.682	18451.682	FALSE
13	15	-425.5	1	-20951.182	20100.182	FALSE
13	16	-21289	0.0353	-41814.682	-763.3181	TRUE
13	17	4671.5	1	-15854.182	25197.182	FALSE
13	18	32721.5	0.0001	12195.818	53247.182	TRUE
13	19	-5992	0.9999	-26517.682	14533.682	FALSE
13	20	231	1	-20294.682	20756.682	FALSE
13	21	317.5	1	-20208.182	20843.182	FALSE
13	22	-9806	0.9558	-30331.682	10719.682	FALSE
13	23	-20955.5	0.0412	-41481.182	-429.8181	TRUE
13	24	-1911.5	1	-22437.182	18614.182	FALSE

13	25	-3022	1	-23547.682	17503.682	FALSE
13	26	1760.5	1	-18765.182	22286.182	FALSE
13	27	9973	0.9482	-10552.682	30498.682	FALSE
13	28	3	1	-20522.682	20528.682	FALSE
14	15	1648.5	1	-18877.182	22174.182	FALSE
14	16	-19215	0.0887	-39740.682	1310.6819	FALSE
14	17	6745.5	0.9996	-13780.182	27271.182	FALSE
14	18	34795.5	0	14269.818	55321.182	TRUE
14	19	-3918	1	-24443.682	16607.682	FALSE
14	20	2305	1	-18220.682	22830.682	FALSE
14	21	2391.5	1	-18134.182	22917.182	FALSE
14	22	-7732	0.9972	-28257.682	12793.682	FALSE
14	23	-18881.5	0.102	-39407.182	1644.1819	FALSE
14	24	162.5	1	-20363.182	20688.182	FALSE
14	25	-948	1	-21473.682	19577.682	FALSE
14	26	3834.5	1	-16691.182	24360.182	FALSE
14	27	12047	0.7791	-8478.6819	32572.682	FALSE
14	28	2077	1	-18448.682	22602.682	FALSE
15	16	-20863.5	0.0429	-41389.182	-337.8181	TRUE
15	17	5097	1	-15428.682	25622.682	FALSE
15	18	33147	0.0001	12621.318	53672.682	TRUE
15	19	-5566.5	1	-26092.182	14959.182	FALSE
15	20	656.5	1	-19869.182	21182.182	FALSE
15	21	743	1	-19782.682	21268.682	FALSE
15	22	-9380.5	0.9716	-29906.182	11145.182	FALSE
15	23	-20530	0.0499	-41055.682	-4.3181	TRUE
15	24	-1486	1	-22011.682	19039.682	FALSE
15	25	-2596.5	1	-23122.182	17929.182	FALSE
15	26	2186	1	-18339.682	22711.682	FALSE
15	27	10398.5	0.9248	-10127.182	30924.182	FALSE
15	28	428.5	1	-20097.182	20954.182	FALSE
16	17	25960.5	0.0036	5434.8181	46486.182	TRUE
16	18	54010.5	0	33484.818	74536.182	TRUE
16	19	15297	0.3752	-5228.6819	35822.682	FALSE
16	20	21520	0.0317	994.3181	42045.682	TRUE
16	21	21606.5	0.0305	1080.8181	42132.182	TRUE
16	22	11483	0.8383	-9042.6819	32008.682	FALSE
16	23	333.5	1	-20192.182	20859.182	FALSE
16	24	19377.5	0.0827	-1148.1819	39903.182	FALSE
16	25	18267	0.1311	-2258.6819	38792.682	FALSE
16	26	23049.5	0.0153	2523.8181	43575.182	TRUE
16	27	31262	0.0002	10736.318	51787.682	TRUE

16	28	21292	0.0353	766.3181	41817.682	TRUE
17	18	28050	0.0013	7524.3181	48575.682	TRUE
17	19	-10663.5	0.9072	-31189.182	9862.1819	FALSE
17	20	-4440.5	1	-24966.182	16085.182	FALSE
17	21	-4354	1	-24879.682	16171.682	FALSE
17	22	-14477.5	0.4729	-35003.182	6048.1819	FALSE
17	23	-25627	0.0043	-46152.682	-5101.3181	TRUE
17	24	-6583	0.9997	-27108.682	13942.682	FALSE
17	25	-7693.5	0.9974	-28219.182	12832.182	FALSE
17	26	-2911	1	-23436.682	17614.682	FALSE
17	27	5301.5	1	-15224.182	25827.182	FALSE
17	28	-4668.5	1	-25194.182	15857.182	FALSE
18	19	-38713.5	0	-59239.182	-18187.818	TRUE
18	20	-32490.5	0.0001	-53016.182	-11964.818	TRUE
18	21	-32404	0.0001	-52929.682	-11878.318	TRUE
18	22	-42527.5	0	-63053.182	-22001.818	TRUE
18	23	-53677	0	-74202.682	-33151.318	TRUE
18	24	-34633	0	-55158.682	-14107.318	TRUE
18	25	-35743.5	0	-56269.182	-15217.818	TRUE
18	26	-30961	0.0003	-51486.682	-10435.318	TRUE
18	27	-22748.5	0.0177	-43274.182	-2222.8181	TRUE
18	28	-32718.5	0.0001	-53244.182	-12192.818	TRUE
19	20	6223	0.9999	-14302.682	26748.682	FALSE
19	21	6309.5	0.9999	-14216.182	26835.182	FALSE
19	22	-3814	1	-24339.682	16711.682	FALSE
19	23	-14963.5	0.4137	-35489.182	5562.1819	FALSE
19	24	4080.5	1	-16445.182	24606.182	FALSE
19	25	2970	1	-17555.682	23495.682	FALSE
19	26	7752.5	0.9971	-12773.182	28278.182	FALSE
19	27	15965	0.3042	-4560.6819	36490.682	FALSE
19	28	5995	0.9999	-14530.682	26520.682	FALSE
20	21	86.5	1	-20439.182	20612.182	FALSE
20	22	-10037	0.945	-30562.682	10488.682	FALSE
20	23	-21186.5	0.037	-41712.182	-660.8181	TRUE
20	24	-2142.5	1	-22668.182	18383.182	FALSE
20	25	-3253	1	-23778.682	17272.682	FALSE
20	26	1529.5	1	-18996.182	22055.182	FALSE
20	27	9742	0.9585	-10783.682	30267.682	FALSE
20	28	-228	1	-20753.682	20297.682	FALSE
21	22	-10123.5	0.9406	-30649.182	10402.182	FALSE
21	23	-21273	0.0356	-41798.682	-747.3181	TRUE
21	24	-2229	1	-22754.682	18296.682	FALSE

21	25	-3339.5	1	-23865.182	17186.182	FALSE
21	26	1443	1	-19082.682	21968.682	FALSE
21	27	9655.5	0.9619	-10870.182	30181.182	FALSE
21	28	-314.5	1	-20840.182	20211.182	FALSE
22	23	-11149.5	0.869	-31675.182	9376.1819	FALSE
22	24	7894.5	0.9963	-12631.182	28420.182	FALSE
22	25	6784	0.9996	-13741.682	27309.682	FALSE
22	26	11566.5	0.8301	-8959.1819	32092.182	FALSE
22	27	19779	0.0696	-746.6819	40304.682	FALSE
22	28	9809	0.9557	-10716.682	30334.682	FALSE
23	24	19044	0.0953	-1481.6819	39569.682	FALSE
23	25	17933.5	0.1496	-2592.1819	38459.182	FALSE
23	26	22716	0.018	2190.3181	43241.682	TRUE
23	27	30928.5	0.0003	10402.818	51454.182	TRUE
23	28	20958.5	0.0411	432.8181	41484.182	TRUE
24	25	-1110.5	1	-21636.182	19415.182	FALSE
24	26	3672	1	-16853.682	24197.682	FALSE
24	27	11884.5	0.797	-8641.1819	32410.182	FALSE
24	28	1914.5	1	-18611.182	22440.182	FALSE
25	26	4782.5	1	-15743.182	25308.182	FALSE
25	27	12995	0.6642	-7530.6819	33520.682	FALSE
25	28	3025	1	-17500.682	23550.682	FALSE
26	27	8212.5	0.9938	-12313.182	28738.182	FALSE
26	28	-1757.5	1	-22283.182	18768.182	FALSE
27	28	-9970	0.9483	-30495.682	10555.682	FALSE
